# Mice defective in interferon signaling help distinguish between primary and secondary pathological pathways in a mouse model of neuronal forms of Gaucher disease

**DOI:** 10.1186/s12974-020-01934-x

**Published:** 2020-09-07

**Authors:** Ayelet Vardi, Shifra Ben-Dor, Soo Min Cho, Ulrich Kalinke, Julia Spanier, Anthony H. Futerman

**Affiliations:** 1 Department of Biomolecular Sciences, Weizmann Institute of Science, 76100 Rehovot, Israel; 2 Life Sciences Core Facilities, Weizmann Institute of Science, 76100 Rehovot, Israel; 3 Current address: NuriScience Inc., Achasan-ro 320, Seoul, 05053 Republic of Korea; 4 TWINCORE-Centre for Experimental and Clinical Infection Research, Institute for Experimental Infection Research, 30625 Hanover, Germany

**Keywords:** Gaucher disease, Pathogen recognition receptors, Type 1 interferon, Neurodegenerative diseases, Lipid metabolism, Lysosomal storage diseases

## Abstract

**Background:**

The type 1 interferon (IFN) response is part of the innate immune response and best known for its role in viral and bacterial infection. However, this pathway is also induced in sterile inflammation such as that which occurs in a number of neurodegenerative diseases, including neuronopathic Gaucher disease (nGD), a lysosomal storage disorder (LSD) caused by mutations in *GBA*.

**Methods:**

Mice were injected with conduritol B-epoxide, an irreversible inhibitor of acid beta-glucosidase, the enzyme defective in nGD. MyTrMaSt null mice, where four adaptors of pathogen recognition receptors (PRRs) are deficient, were used to determine the role of the IFN pathway in nGD pathology. Activation of inflammatory and other pathways was analyzed by a variety of methods including RNAseq.

**Results:**

Elevation in the expression of PRRs associated with the IFN response was observed in CBE-injected mice. Ablation of upstream pathways leading to IFN production had no therapeutic benefit on the lifespan of nGD mice but attenuated neuroinflammation. Primary and secondary pathological pathways (i.e., those associated or not with mouse survival) were distinguished, and a set of ~210 genes including those related to sphingolipid, cholesterol, and lipoprotein metabolism, along with a number of inflammatory pathways related to chemokines, TNF, TGF, complement, IL6, and damage-associated microglia were classified as primary pathological pathways, along with some lysosomal and neuronal genes.

**Conclusions:**

Although IFN signaling is the top elevated pathway in nGD, we demonstrate that this pathway is not related to mouse viability and is consequently defined as a secondary pathology pathway. By elimination, we defined a number of critical pathways that are directly related to brain pathology in nGD, which in addition to its usefulness in understanding pathophysiological mechanisms, may also pave the way for the development of novel therapeutic paradigms by targeting such pathways.

## Background

The lysosomal storage disease (LSD), Gaucher disease (GD), is caused by mutations in *GBA*, which encodes the lysosomal hydrolase, acid beta-glucosidase (GCase). GD is divided into neuronopathic (types 2 and 3) (nGD) and non-neuronopathic forms (type 1), depending on the involvement of symptoms associated with the central nervous system [1, 2]. Little is known about pathological mechanisms that lead to brain disease, but among these is neuroinflammation. Surprisingly, among the inflammatory pathways [3], activation of the type 1 interferon (IFN) response was demonstrated [4] in Gba^flox/flox^ (nestin-Cre mice) in which GCase deficiency in the brain is restricted to cells of neuronal lineage, with microglia displaying normal GCase levels [5]. In an unbiased gene profile analysis, genes associated with the type 1 IFN-related pathway were highly upregulated [4].

The IFN response is normally considered to occur as a result of pathogen recognition receptor (PRR) stimulation. The PRRs are part of the innate immune system and respond to either pathogen-associated molecular patterns (PAMPs) or to endogenous molecules secreted following tissue stress or injury, known as danger-associated molecular patterns (DAMPs) [6]. Several classes of PRRs are known, and a number can trigger the IFN response. The most well-defined PRRs are the Toll-like receptors (TLRs); after ligand binding, TLRs dimerize, the Toll/IL-1 receptor (TIR) domain associates with TIR domain-containing adaptor proteins, such as myeloid differentiation factor 88 (MyD88) and TIR domain-containing adaptor protein-inducing IFN-β (TRIF), which initiates downstream signaling [7]. The Nod-like receptor, NOD2, the RIG-I receptor retinoic acid-inducible gene I (RIG-I), and the melanoma differentiation-associated factor 5 (MDA5) use mitochondrial antiviral signaling protein (MAVS) as their adaptor [8]. The most important IFN-inducing cytosolic DNA sensing pathways is the cyclic guanosine monophosphate-adenosine monophosphate synthase (cGAS), stimulator of IFN gene (STING, *TMEM173*) axis. Upon binding of DNA, cGAS catalyzes the formation of the secondary messenger, 2′,3′-cyclic guanosine monophosphate-adenosine monophosphate (cGAMP), which binds STING and subsequently activates an antiviral cytokine response [9].

In the current study, we have examined the effect of inducing nGD, using a chemical inhibitor (conduritol B-epoxide, CBE [10, 11]), in a quadrat-deficient mouse with a combined deficiency of TLR, RIG-I like receptor (RLR) and STING signaling (*Myd88*^*-/-*^*,Trif*^*-/-*^*,Mavs*^*-/-*^*,Tmem173*^*-/-*^), referred to as the MyTrMaSt mouse [12]. These are the main PRRs that induce a type 1 IFN response, although it should be noted that IFN can also be induced by other PRRs such as cGAS [12]. We demonstrate that the IFN pathway is not the initial cause for pathology in nGD, but rather a secondary pathological pathway since the lifespan of MyTrMaSt null mice was not altered upon CBE injection compared with wild-type (WT) mice. We then go on to define a subset of genes and pathways as primary pathological pathways which may be responsible for the pathogenesis of nGD.

## Methods

### Mice

MyTrMaSt null mice were generated as described [12]. From postnatal day 8, C57BL/6JOlaHsd mice (Envigo Laboratories, Israel) or MyTrMaSt null mice were injected intraperitoneally (i.p.) daily with 25, 32, 37.5, or 100 mg CBE (Calbiochem Millipore, Darmstadt, Germany) per kilogram body weight, or with phosphate buffered saline (PBS). Genotyping was performed by PCR using genomic DNA extracted from mouse tails. Mice were maintained in the experimental animal center of the Weizmann Institute of Science in specific pathogen-free conditions. All animal experiments were approved by the Weizmann Institute Institutional Animal Care and Use Committee.

### RNA extraction and quantitative PCR

Mice were euthanized using CO_2_ and brains were rapidly removed. Total RNA from half brain was isolated using the RNeasy mini kit (Qiagen GmbH, Hilden, Germany) according to manufacturer’s instructions. cDNA synthesis was performed using a qScript cDNA synthesis kit (Quanta Biosciences, Gaithersburg, MD, USA). Quantitative PCR (qPCR) was performed using the PerfeCT SYBR Green FastMix (Quanta BioSciences, Gaithersburg, MD, USA) and an ABI Prism 7300 Sequence Detection System (Applied Biosystems, Foster City, CA, USA). The primer concentration was 20 nM in a reaction volume of 10 μl. Primer sequences are listed in Additional file 1. IFNα2, TNFα, and IL1β primers were purchased from Quantitect (Qiagen). Each reaction was performed in duplicate. The ΔΔCt method was used to calculate relative changes in gene expression, with hypoxanthine phosphoribosyltransferase 1 (HPRT1) used as a housekeeping gene [13]. *p* values were calculated using a two-tailed, two-independent sample Student’s *t* test.

### Western blotting

Brain tissue was lysed in lysis buffer (50 mM Tris HCl pH 7.6, 150 mM NaCl, 1% NP-40, 0.5% sodium deoxycholate, and 0.1% sodium dodecyl sulfate (SDS)) supplemented with a protease inhibitor mixture (1:100, Sigma-Aldrich) using a GentleMACS dissociator (Miltenyi Biotec, Bergisch Gladbach, Germany). Following homogenization, samples were centrifuged at 14,000 × *g*_av_ for 10 min at 4 °C, and the supernatants collected. Protein was quantified using the BCA protein assay reagent. Seventy micrograms of protein in the sample buffer was electrophoresed on a 10% SDS-polyacrylamide gel and transferred to a nitrocellulose membrane. Blots were incubated with the following primary antibodies: rat anti-MAC2 (1:1000, Cedarlane, Ontario, Canada), mouse anti-tubulin (1:5000, Santa Cruz, Dallas, TX, USA), followed by a horseradish peroxidase-conjugated secondary antibody. Bound antibodies were detected using the Westar Chemiluminescent substrate (Cyanagen, Bologna, Italy). Western blots were analyzed by densitometry and values quantified using a ratio of Mac2 to Tubulin.

### RNAseq

Brain tissue was homogenized using a GentleMACS dissociator and mRNA isolated using the RNeasy mini kit. RNA concentrations (260/230- and 260/280-nm ratios) were measured using a NanoDrop ND-1000 (Thermo Scientific, Waltham, MA, USA). RNA integrity was evaluated using an RNA screen tape on a Tapestation 2200 (Agilent, Santa Clara, CA, USA). A bulk variation of MARSseq [14] was used to construct RNAseq libraries. Sequencing was performed using an Illumina Nextseq-500 75 cycle high output kit (Illumina, San Diego, CA, USA; paired end sequencing). Raw reads were mapped to the *Mus musculus* genome (mm10) using hisat (version 0.1.6). Only reads with unique mapping were considered for further analysis. Differentially expressed genes (DEGs) were selected using a 2-fold change cutoff between two populations and adjusted *p* value for multiple gene testing of < 0.05 [15]. Principle component analysis (PCA) of all DEGs and heatmaps were generated using Partek Genomics Suite® software, version 7.0 (St. Louis, MO, USA), or RStudio (Integrated Development for R. RStudio, Inc., Boston, USA). The BioVenn tool [16] was used to identify common and exclusively expressed genes between groups. Pathway analysis was done using Metascape [17] or Gene Analytics [18].

## Results

### Elevation of PRR expression in nGD mice

Genes encoding various PRRs were elevated in the brain of a genetic model of nGD [4], and we now show a similar elevation in PRR mRNA levels in a chemically induced nGD mouse. Upon injection of C57BL/6JOlaHsd mice with a high dose of CBE (100 mg/kg body weight [10]) for 10 days, a significant elevation in the expression of PRR family members related to IFN signaling was observed, with the highest elevation in a cell surface TLR, *Tlr2* (Table 1). *Tlr1* and *Tlr4*, which are also located on the cell surface, were also significantly elevated. Levels of expression of endosomal TLRs [19], *Tlr3*, *Tlr7*, *Tlr8, Tlr9*, and *Tlr13* were likewise elevated. The elevation of *Tlr* expression implicates signaling via their downstream adaptors, namely MyD88 (used for all TLRs except TLR3) and TRIF (required for TLR3 and TLR4). NOD-like receptor (NLR) family members, such as NOD2, which utilizes the adaptor protein MAVS, can also induce IFN production [20], as can members of the RLR family, RIG-I and MDA5, which also use MAVS. STING, which functions both as a PRR and as a signaling adaptor, induces expression of type 1 IFN via the NFκB and IRF3 pathways [21]. All of these PRRs were elevated (Table 1), suggesting that GlcCer accumulation, or a downstream effector, acts as a DAMP.

**Table 1:** mRNA levels of genes encoding PRRs in brain homogenates from 18-day-old mice injected daily with 100 mg/kg body weight CBE from day 8

Gene	Fold-change CBE *versus* PBS	*p* value
*Tlr1*	7.5 ± 1.4	< 0.001
*Tlr2*	17.3 ± 2.8	< 0.005
*Tlr3*	5.0 ± 1.4	< 0.005
*Tlr4*	4.1 ± 0.5	< 0.001
*Tlr5*	1.7 ± 0.6	n.s.
*Tlr6*	3.7 ± 2.0	n.s.
*Tlr7*	6 ± 1.8	< 0.001
*Tlr8*	3.8 ± 2.6	n.s.
*Tlr9*	2.4 ± 0.9	n.s.
*Tlr11*	1.3 ± 1.1	n.s.
*Tlr12*	1.1 ± 0.4	n.s.
*Tlr13*	9.1 ± 1.5	< 0.001
*Rig-I*	6.6 ± 1.5	< 0.005
*Mda5*	8.4 ± 2.3	< 0.005
*Nod2*	2.7 ± 0.8	< 0.05
*cGAS*	3.1 ± 0.6	0.005
*Sting*	2.8 ± 0.8	< 0.05

Results are expressed as fold-change of CBE-*versus* PBS-injected mice (*n* = 3) and are means ± SEM

*n.s.* not significant

### The IFN response does not play a role in nGD mouse lifespan

**Fig. 1:**
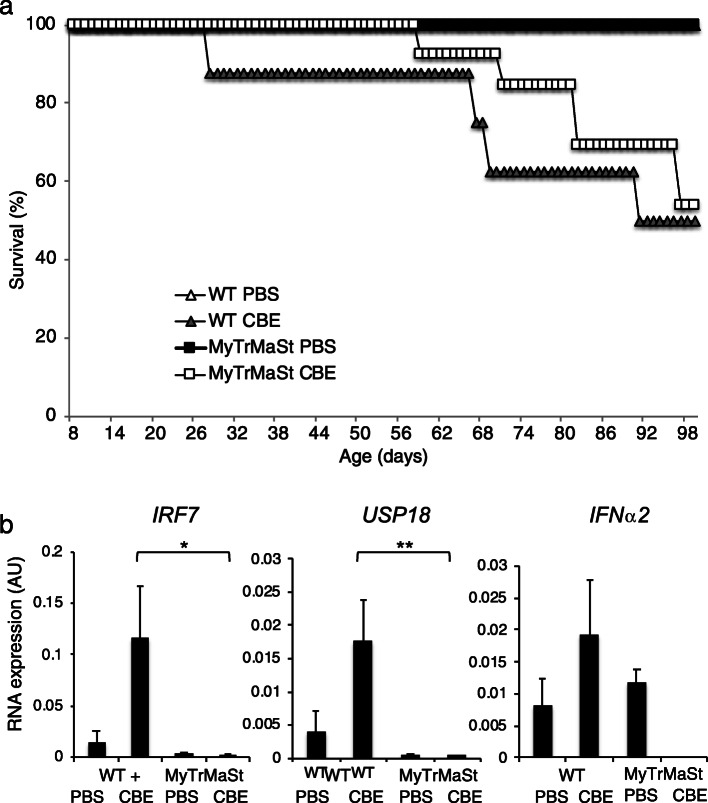
Inhibition of the type 1 IFN response has no effect on lifespan. **a** Kaplan-Meier survival curve of WT mice injected i.p. with PBS (*n* = 4) or 25 mg/kg CBE (*n* = 8) or MyTrMaSt null mice injected with PBS (*n* = 6) or 25 mg/kg CBE (*n* = 13). **b** RT-PCR from brain homogenates (day 96) of WT and MyTrMaSt null mice injected with PBS (*n* = 2) or CBE (25 mg/kg) (*n* = 4). Cycle threshold values were normalized to levels of hypoxanthine phosphoribosyltransferase 1 (HPRT1). * *p* < 0.05, ** *p* < 0.01

We previously demonstrated that ablation of the type 1 IFN receptor (IFNAR1) attenuated neuroinflammation but had no effect on nGD mouse viability [4]. IFN is nevertheless secreted in IFNAR null mice. To determine the effect of inhibiting pathways upstream to IFNAR, we injected MyTrMaSt null mice with a low dose of CBE (25 mg/kg, since higher doses (i.e., 100 mg/kg CBE) cause extremely severe disease, with mice not surviving beyond 20 days of age [10]. The mean lifespan of MyTrMaSt null mice was similar to that of WT mice injected with CBE (Fig. 1a) although there was a significant variability between individual mice. Likewise, no effect was seen on the lifespan using higher doses [10] of CBE (32 mg/kg and 37.5 mg/kg). Levels of expression of *Irf7*, *Usp18*, and *Ifnα*, which play critical roles in IFN signaling, were elevated upon CBE injection in WT mice, as previously observed [4], but not elevated in MyTrMaSt null mice upon CBE injection (Fig. 1b). This demonstrates that the IFN pathway is not required for nGD pathology.

### Attenuated neuroinflammation in MyTrMaSt null mice

**Fig. 2:**
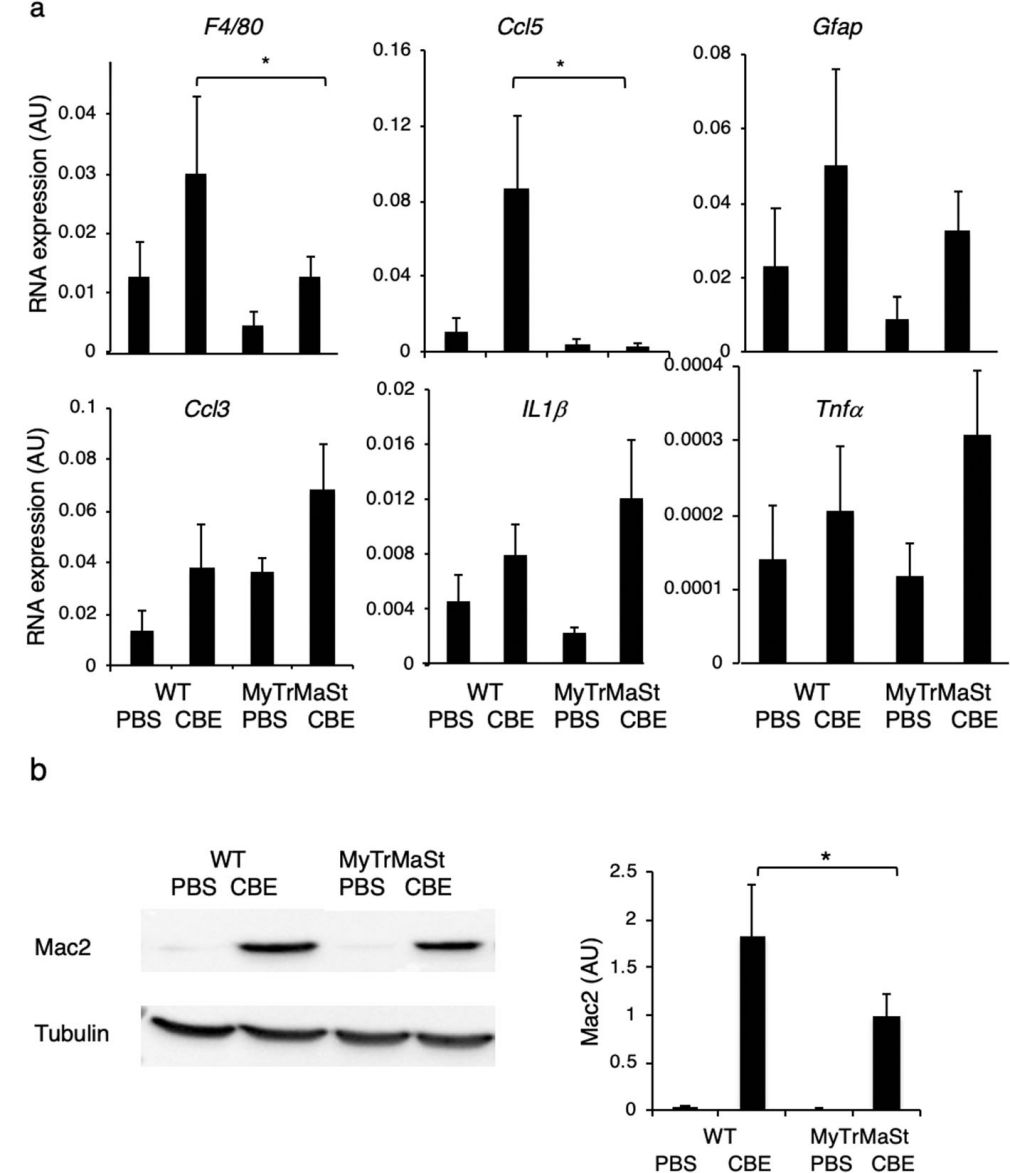
Attenuated neuroinflammation in MyTrMaSt null mice. **a** mRNA levels in brain homogenates from 96–130-day-old CBE-injected mice (25 mg/kg; *n* = 6–11) and PBS injected mice (*n* = 4–6). RNA expression is shown as arbitrary units (AU). * *p* < 0.05. **b** A representative western blot from brain homogenates is shown (70 μg of protein) of 19-day-old WT or MyTrMaSt null mice injected with 100 mg/kg CBE or PBS from 8 days of age. Results are representative of 3 experiments for control mice and 5 for CBE-treated mice, which all gave similar results. Tubulin was used as loading control and was similar in each lane. Quantification is normalized to tubulin. *p < 0.05

We next determined the relationship between the PRR pathway and components of inflammatory mediators that are altered in nGD such as chemokines, Il1β, and TNFα [22]. Levels of *F4/80*, which is expressed on microglia and macrophages, as well as the inflammatory chemokine *Ccl5* (*Rantes*), were significantly reduced in MyTrMaSt null mice injected with 25 mg/kg CBE compared with control mice injected with CBE, but in contrast, no differences were observed in levels of *Gfap*, *Ccl3* (*Mip1α*), *IL1β*, and *TNFα* (Fig. 2a) even by using higher doses of CBE (32 mg/kg and 37.5 mg/kg; data not shown). Likewise, the main inducers of the IFN pathway did not affect levels of an activated astrocyte marker (*Gfap*), which remained elevated when MyTrMaSt null mice were injected with doses of CBE as high as 100 mg/kg body weight (Fig. 2a, b). Levels of *Mac2*, which is expressed by cells of the macrophage/microglia lineage (Fig. 2b), were reduced in CBE-injected MyTrMaSt null mice similar to the reduction in *F4/80*. Since levels of macrophage/microglia markers (*F4/80, Mac2*) and the inflammatory marker *Ccl5* were reduced in response to the inhibition of the IFN response, while levels of other inflammatory markers (*Ccl3, IL1β*, *TNFα*) and an astrocyte marker (*Gfap*) remain unchanged, this suggests that some pathways of neuroinflammation are affected upon loss of the IFN response, while others are not.

### RNAseq distinguishes between primary and secondary pathological pathways

To distinguish between genes involved in pathophysiology that are associated with mouse death (which we define as “primary pathological pathways”) and those that are unrelated to mouse death (“secondary pathological pathways”), we performed RNAseq [MARSseq, [14]] on brain homogenates from four groups of mice, namely WT mice injected with PBS (referred to as *WT + PBS*), WT mice injected with 100 mg/kg body weight CBE (*WT + CBE*), MyTrMaSt null mice injected with PBS (*MyTrMaSt + PBS*) and MyTrMaSt null mice injected with 100 mg/kg body weight CBE (*MyTrMaSt + CBE*). Comparison of DEGs in WT mice injected with CBE *versus* PBS, compared with MyTrMaSt null mice injected with CBE *versus* PBS should distinguish between genes associated with putative primary or secondary pathological pathways.

**Fig. 3:**
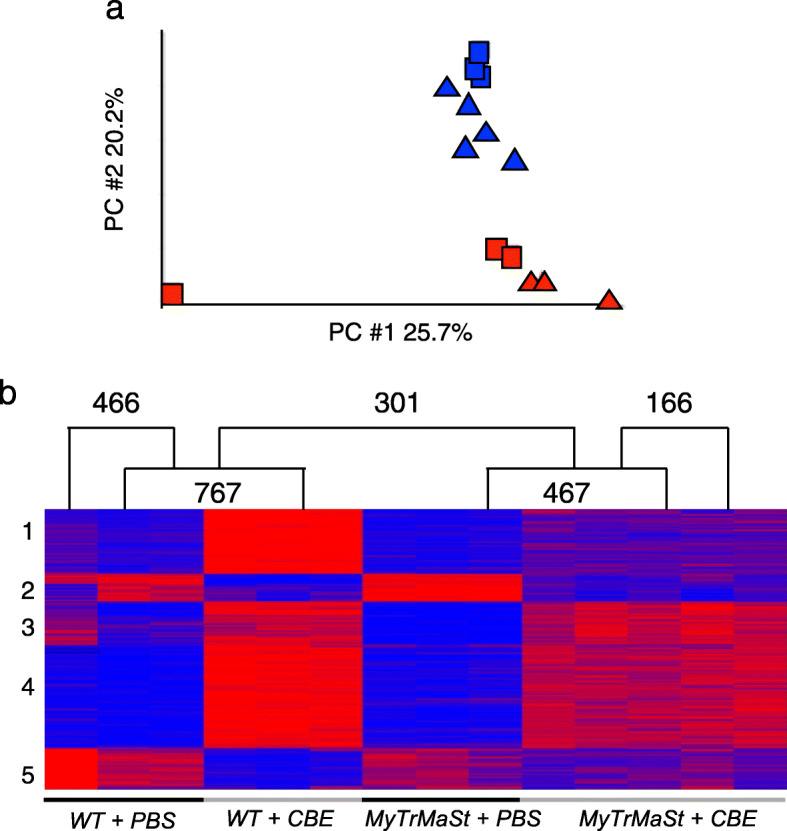
RNAseq discriminates three groups of genes. **a** PCA of DEGs in *WT + PBS* (*n* = 3) (*red squares*) versus *WT + CBE* (*n* = 3) (*blue squares*) and *MyTrMaSt + PBS* (*n* = 3) (*red triangles*) versus *MyTrMaSt + CBE* (*n* = 5) (*blue triangles*); fold-change > 2, *p* adjusted < 0.05. **b** K-means clustering of all DEGs (fold-change > 2, *p* adjusted < 0.05). *Red* (high, > 1) and *blue* (low, less than − 1) in the heat map represent relative gene expression. Five clusters are indicated

A total of 13,866 genes were detected by RNAseq. Principle component analysis (PCA) revealed that PBS-injected mice clearly separate from CBE-injected mice, with the exception of one outlier WT mouse (Fig. 3a). A total of 767 genes were differentially expressed (DE) (fold change ≥ 2, *p* adjusted < 0.05) in *WT + CBE* versus *WT + PBS* (599 upregulated and 168 downregulated), and 467 genes were DE in *MyTrMaSt + CBE* versus *MyTrMaSt + PBS* (392 upregulated, 75 downregulated) (Fig. 3b). K-means clustering of the DEGs (Fig. 3b) indicated 5 gene clusters. The 215 genes in cluster 1 were elevated only in the *WT + CBE* group. The genes in clusters 2 (93 genes) and 5 (138 genes) display similar decreased expression in the two CBE-treated groups. The genes in clusters 3 and 4 (143 and 345 genes) displayed reduced expression in *MyTrMaSt + CBE* compared with the *WT + CBE* group. Comparison of the DEGs in the *WT + CBE* group versus *WT* + *PBS* with our previous high-throughput studies [4, 23] indicated similar changes in gene expression, even though previous studies were performed on different days of CBE injection and in different brain areas.

Analysis of DEGs in *WT + CBE* versus *WT* + *PBS* and in *MyTrMaSt + CBE* versus *MyTrMaSt + PBS* indicated 466 DEGs (767 − 301, Fig. 3b) in the *WT+CBE* versus *WT + PBS* group (mainly in cluster 1), whereas there are 301 DEGs common to the two groups (mainly in clusters 3 and 4). A total of 166 genes (467 − 301, Fig. 3b) are DE only in *MyTrMaSt + CBE* versus *MyTrMaSt + PBS* (clusters 2 and 3) (Fig. 3b). We define the group of 466 DEGs as associated with secondary pathological pathways, since the expression of these genes is altered in WT mice, whereas the 301 DEGs common to the two groups of CBE-injected mice are defined as primary pathological pathways, since the lifespan of the MyTrMaSt null mice is similar to WT mice injected with CBE.

**Fig. 4:**
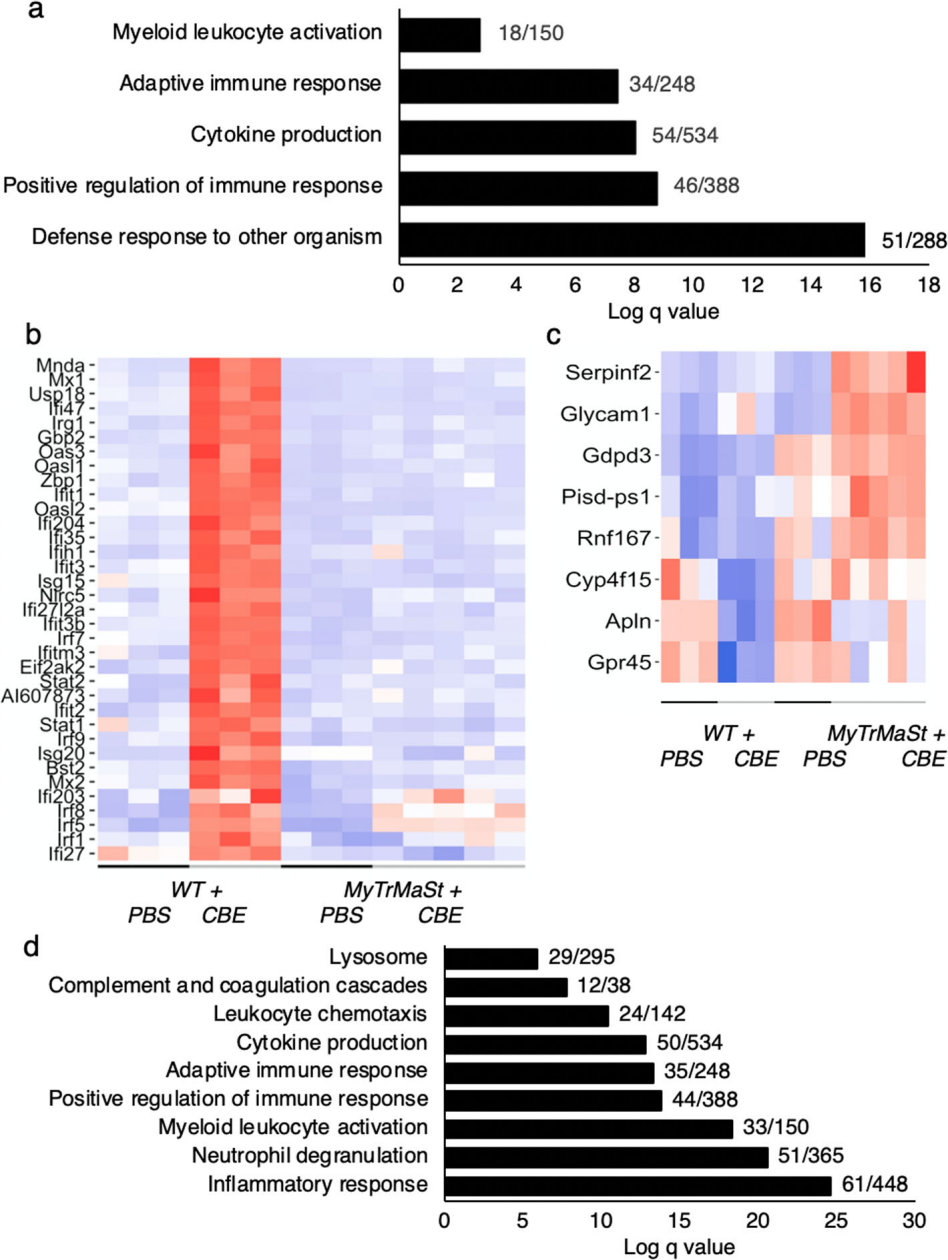
Loss of IFN signaling in MyTrMaSt null mice. **a** Pathway analysis of the 466 DEGs in *WT + PBS* versus *WT + CBE*. Numbers of identified genes out of the total genes associated with each pathway are shown. **b** Heatmap of representative IFN genes. *Red* (high, > 1) and *blue* (low, < 1) in the heat map represent relative gene expression. Some of the DEGs were validated by PCR; *WT + CBE* versus *WT + PBS*, fold-change 27.5 ± 2.9 (*Irf7*), 168.4 ± 27.2 (*Irg1*); *MyTrMaSt + CBE* versus *MyTrMaSt + PBS*, fold-change 1.0 ± 0.1 (*Irf7*), 2.3 ± 0.2 (*Irg1*). **c** Heatmap of the 8 genes upregulated in *MyTrMaSt null + CBE* versus *WT + CBE* mice. Some of the DEGs were validated by PCR: *MyTrMaSt + CBE* versus *WT + CBE*, fold-change 2.35 ± 0.3 (*Serpinf2*), 1.93 ± 0.3 (*Glycam1*), 1.86 ± 0.2 (*Apln*). **d** Pathway analysis of the 301 common DEGs. Numbers of identified genes out of the total genes associated with each pathway are shown

Analysis of the 466 DEGs associated with secondary pathological pathways demonstrated enrichment in genes related to the type 1 IFN response (Fig. 4a) [4], with 8 of the 10 most upregulated genes induced by IFN. As expected, these genes were not DE in MyTrMaSt null mice (Fig. 4b). Various cytokines, chemokines, and *Tnfα*-induced genes were also enriched in the 466 genes (Fig. 4a). Of the 466 DEGs, approximately one third (149) were downregulated. Based on single-cell RNAseq analyses [24, 25], 42% of the downregulated genes can be assigned to neurons, consistent with neuronal loss by a “dying-back” mechanism in nGD [26, 27]. Among the downregulated genes in *WT + CBE versus WT + PBS* are *Mapre3*, *Map1lc3a*, and *Mast2* microtubule genes, a pathway which underlies neurodegeneration via a dying-back mechanism [28]. The relevance of the 166 DEGs (110 upregulated and 56 downregulated) in *MyTrMaSt + CBE* versus *MyTrMaSt + PBS* mice (Fig. 3b) is somewhat ambivalent, particularly as a number of the DEGs showed a similar fold-change as in WT mice injected with CBE, but did not reach statistical significance (see clusters 2 and 3, Fig. 3b). Only 8 genes were exclusively upregulated in *MyTrMaSt + CBE* versus *WT + CBE* (Fig. 4c), and no obvious connection between them could be ascertained. Of the 56 downregulated genes, 27% are expressed in neurons.

Since MyTrMaSt null and WT mice displayed a similar life span when injected with CBE, the common 301 DEGs in both CBE-injected groups are likely to be associated with primary pathological pathways. Pathway analysis of the 301 DEGs revealed changes in the lysosome, complement cascades and in a variety of immune responses (Fig. 4d). However, it was possible to further divide the 301 DEGs in two groups, ~90 genes that differed more than 2-fold between MyTrMaSt and WT upon CBE injection (Table 2) and 210 genes whose levels are essentially similar (< 2-fold change) (Table 3). The ~90 genes are unlikely to be primary pathological components and encompass a number of pathways (Table 2). Thus, a number of chemokines and microglia genes were significantly lower in MyTrMaSt null mice compared with WT mice, as were genes associated with TNFα and TGFβ pathways. Expression of a number of cathepsin genes were also lower, as were some, but not all genes associated with complement, DAMs, and cholesterol metabolism. A number of genes associated with lipoproteins were elevated in *WT + CBE* mice but reduced in *MyTrMaSt + CBE*.

**Table 2:** DEGs in WT and MyTrMaSt mice

Gene	*WT + CBE* versus *WT + PBS*	*MyTrMaSt + CBE* versus *MyTrMaSt + PB*	
	Fold-change	Fold-change	
Cholesterol metabolism			
*Dhcr24*	*−* 3.34	*− 1.67*	
*Mvd*	*−* 2.60	*− 1.27*	
*S1pr3*	12.0	4.20	
Lipoprotein metabolism			
*Apobec1*	13.0	3.63	
*Apobec3*	6.70	2.50	
*Pon3*	7.34	3.30	
Metalloproteinases			
*Aspg*	22.2	8.50	
*Mmp19*	9.80	4.20	
*Timp1*	60.4	13.9	
Lysosome			
*Ctsc*	8.60	2.60	
*Ctss*	6.60	2.90	
Inflammation (chemokines)			
*A2m*	17.3	7.41	
*Ccl2*	161	14.1	
*Ccl5*	29.3	*− 1.02*	
*Ccl12*	39.6	8.80	
*Cxcl1*	42.2	16.1	
*Cxcl10*	77.7	3.06	
*Cxcl16*	12.6	3.38	
*Ptprc*	12.4	2.40	
Inflammation (TNF)			
*Ptx3*	92.2	8.04	
*Steap4*	51.4	3.90	
*Tnfaip2*	40.6	4.07	
Inflammation (TGF)			
*Gdf15*	59.9	4.80	
*Tgfbi*	9.63	2.05	
Inflammation (Microglia)			
*Cd300lf*	42.3	11.4	
*Lgals3bp*	13.9	4.35	
*Mac2*	60.1	21.8	
*P2ry6*	7.96	3.96	
Complement			
*C3*^*1*^	32.1	3.01	
*C3ar1*	80.6	5.60	
*Itgb2*	14.7	5.78	
Others			
*Cst7*	102	37.9	
*Serpina3n*	57.8	17.1	
*Cd5l*	32.6	7.9	

A selection of the 90 DEGs whose levels were reduced > 2-fold in MyTrMaSt mice compared with WT mice, but were nevertheless still DE between *MyTrMaSt + CBE* versus *MyTrMaSt + PBS*. Fold-changes were all statistically significant (*p* < 0.01) except for the values in italics

*n.s.* not significant

^1^ Validated by PCR: *WT + CBE* versus *WT + PBS*, fold-change 80.7 ± 16.2 (*C3*). *MyTrMaSt + CBE* versus *MyTrMaSt + PBS*, fold-change 21.2 ± 8.6

**Table 3:** Genes associated with primary neuropathological pathways

Gene	*WT + CBE* versus *WT + PBS*	*WT + CBE* versus MyTrMaSt + PBS	CBE + SRT
	Fold-change	Fold-change	
SL metabolism			
*Ugt8a*	− 3.46	− 3.97	++
*Fa2h*	− 2.00	− 2.00	++
*Gal3st1*	− 2.42	− 1.33	n.s.
Cholesterol metabolism			
*Msmo1*	− 2.28	− 2.27	++
*Ch25h*	20.3	12.8	+
*Dhcr7*	− 2.78	− 1.62	n.s.
*Tm7sf2*	− 2.05	− 1.44	n.s.
*Hmgcs1*	− 2.63	− 2.07	n.s.
*Fdps*	− 2.90	− 2.10	n.s.
Lipoprotein metabolism and lipid droplets			
*Apoc1*	2.76	2.42	++
*Plin2*^*1*^	5.57	2.90	++
*Plin4*	9.97	41.3	++
*Apoe*	1.85	1.56	+
Metalloproteinases			
*Adam8*	6.20	5.20	+
*Mt1*^*1*^	3.80	3.40	++
*Mt2*	6.30	4.80	++
Lysosome			
*Gusb*^*1*^	6.08	5.88	n.s.
*Hexb*	3.43	3.17	+
*Naglu*	2.60	2.27	+
*CtsD*^*1*^	4.48	4.68	+
*Ctsz*	6.62	5.30	+
*Galns*	2.88	2.39	n.s.
Neuronal genes			
*Ccdc160*	− 2.33	− 2.11	n.s.
*Glra1*	− 2.04	− 2.63	n.s.
*Srpk1*	− 2.45	− 2.05	n.s.
Inflammation (chemokines)			
*Ccl3*	39.7	55.5	+
*Ccl4*	49.2	32.7	+
*Cx3cr1*^*1*^	3.70	2.10	n.s.
Inflammation (TNF)			
*Tnfrsf1a*	3.02	4.68	+
*Tnfaip8l2*	4.08	4.02	+
*Slamf9*	16.1	15.0	+
*Ltbr*	2.33	3.08	n.s.
*Litaf*	3.32	2.32	n.s.
Inflammation (TGF)			
*Tgfbr2*	2.02	2.81	n.s.
*Cd109*	8.53	10.4	++
Inflammation *(IL6)*			
*Il6ra*	5.03	3.08	n.s.
*Osmr*	4.73	9.70	+
Inflammation (Microglia)			
*Lyz2*	13.2	7.80	+
*Mpeg1*	9.07	11.5	+
*Cd68*	8.08	13.9	+
Inflammation (Astrocytes)			
*Agt*	2.10	2.80	n.s.
*Gfap*	9.86	9.69	+
Complement			
*C1qa*	4.16	4.61	+
*C1qb*	5.55	5.11	+
*C1qc*	7.53	5.17	+
*C4b*	7.35	7.08	+
*C5ar1*^*1*^	27. 7	14.8	+
DAMs			
*Trem2*	6.12	8.88	+
*Tyrobp*^*1*^	6.65	6.55	+
*Cd63*	3.70	3.20	+
*Clec7a*	43.9	31.3	+
*Csf1*	3.14	2.20	-
*Itgax*	61.1	39.6	-
Others			
*St14*	3.28	4.85	-
*Mrpl35*	− 2.33	− 2.03	n.s.

Genes whose levels were changed < 2-fold in MyTrMaSt mice compared with WT mice. Fold-changes were all statistically significant (*p* < 0.01). *The right-hand* column indicates genes that reverted to control levels upon SRT (from Blumenreich et al., submitted for publication; see Discussion).

*n.s.* not significant

^1^ Validated by PCR: *WT + CBE* versus *WT + PBS*, fold-change 7.9 ± 3.1 (*Plin2*), 1.8 ± 0.2 (*Mt1*), 5.9 ± 1.4 (*Gusb*), 5.9 ± 1.4 (*CtsD*), 3.1 ± 0.6 (*Cx3cr1*), 31.4 ± 3.8 (*C5ar1*), 11.4 ± 3.0 (*Tyrobp*). *MyTrMaSt + CBE* versus *MyTrMaSt + PBS*, fold-change 4.1 ± 1.3 (*Plin2*), 1.9 ± 0.2 (*Mt1*), 6.7 ± 0.5 (*Gusb*), 5.2 ± 0.5 (*CtsD*), 3.2 ± 0.4 (*Cx3cr1*), 15.9 ± 1.2 (*C5ar1*), 9.1 ± 0.5 (*Tyrobp*)

+ Genes whose expression were reduced upon SRT, yet remained upregulated

++ Genes that reverted to control levels upon SRT

- Genes whose expression was not affected upon SRT

The most interesting set of genes are the 210 genes that were unchanged (< 2-fold) in MyTrMaSt mice compared with WT mice injected with CBE (Table 3). Although some of these genes are associated with similar pathways as in Table 2, they are nevertheless quite distinct. For instance, levels of expression of three biosynthetic genes associated with sphingolipid (SL) metabolism remain downregulated in MyTrMaSt null mice, indicating that blocking the IFN response does not revert or change the defects in SL metabolism, which is perhaps not surprising since GD is a SL LSD; likewise, genes associated with the lysosome were also unaltered. More unexpectedly, genes associated with cholesterol metabolism and lipoprotein metabolism were also unaltered; importantly, *Plin4*, which is found on lipid droplets [29], was one of the few genes whose expression was significantly elevated in MyTrMaSt null mice injected with CBE compared with WT mice injected with CBE. Thus, we conclude that primary pathological pathways include those associated with SLs, lipids, lipoproteins, and lysosomes, which is consistent with the known etiology of nGD.

In addition, and in line with data shown in Fig. 2, inflammation unrelated to IFN pathways is a key component in nGD pathology. Thus, expression of a variety of genes encoding chemokines and pathways associated with TNFα, TGFβ, and IL6 remain elevated in MyTrMaSt null mice, which is consistent with enhanced expression levels of genes associated with both microglia and with astrocytes (such as *Gfap*), supporting the notion that abrogating the IFN response does not attenuate key pathways in neuroinflammation related to nGD symptoms. This is supported by the elevated level of some (but not all, Table 2) components of the complement pathway (Table 3) and DAMs, including *Trem2* and *Tyrobp*. Finally, three neuronal genes may play a critical role in nGD pathology since their levels were also similar in WT and in MyTrMaSt null mice injected with CBE, consistent with the unaltered levels of some metalloproteinases, of which at least one (*Adam8*) is involved in neurodegeneration [30, 31].

## Discussion

Further to our recent study suggesting that the IFN pathway does not play a critical role in nGD pathology [4], we have now taken advantage of the availability of the MyTrMaSt null mouse, which has a deficiency of TLR, RLR, and STING, to differentiate between primary and secondary pathological pathways in nGD. Since the lifespan of MyTrMaSt mice was similar to that of WT mice injected with CBE, even though no IFNs were detected, pathways associated with PRRs and the IFN response cannot be directly involved in pathophysiology of the nGD brain.

Even though we are able to exclude a role for the IFN pathway in primary pathology, components of this pathway are nevertheless the top elevated pathway in the nGD brain and are also elevated in a number of other LSDs, including mucolipidosis type IV and Krabbe disease [32, 33], along with a number of other unrelated neuroinflammatory disorders [34]. This raises the question of how this pathway is activated. One possibility, at least in the three LSDs mentioned above, is that changes in membrane lipid composition result in the activation of PRRs such as TLRs, which upon dimerization associate with adaptor proteins, such as MyD88 and TRIF, to initiate downstream signaling [7]. Receptor dimerization is affected by membrane lipid composition [35]. In GD, changes in lipid composition affect a number of biophysical properties of membranes [36], and we suggest that altered GlcCer levels may directly impinge upon, and perhaps stimulate PRR dimerization, thus activating the IFN response. Nevertheless, since this response can be eliminated with no effect on the lifespan, at least in nGD mice, activation of the IFN pathway may be unrelated to primary disease pathophysiology. Having said that, a recent study has shown that activation of the IFN response in the nGD brain confers resistance to infection by neurotropic viruses (Melamed et al., submitted for publication).

By a process of elimination, we were able to cut down the number of genes associated with primary pathological pathways to ~210, by considering only genes whose expression changed consistently in both WT and MyTrMaSt mice injected with CBE, each compared with its respective PBS-injected control (Table 3). In addition, we have recently completed a study (Blumenreich et al., submitted for publication) in which WT mice were injected with CBE along with a small molecule inhibitor of the critical enzyme in the SL biosynthetic pathway, namely GlcCer synthase (UDP-glucose ceramide glucosyltransferase), in an approach known as substrate reduction therapy (SRT) [37]. SRT led to a decrease in levels of GlcCer and of GlcSph, the two lipids that accumulate in nGD, along with a significant extension of mouse lifespan. Analysis of gene expression by RNAseq revealed that SRT largely reversed the changes in genes and pathways that were DE upon CBE injection, suggesting that these pathways play a vital role in the mouse lifespan and thus pathophysiology, including pathways of GSL metabolism, lipoproteins, and other lipid metabolic pathways, lipid droplets, astrocyte activation, neuronal function, and to some extent, neuroinflammation.

**Fig. 5:**
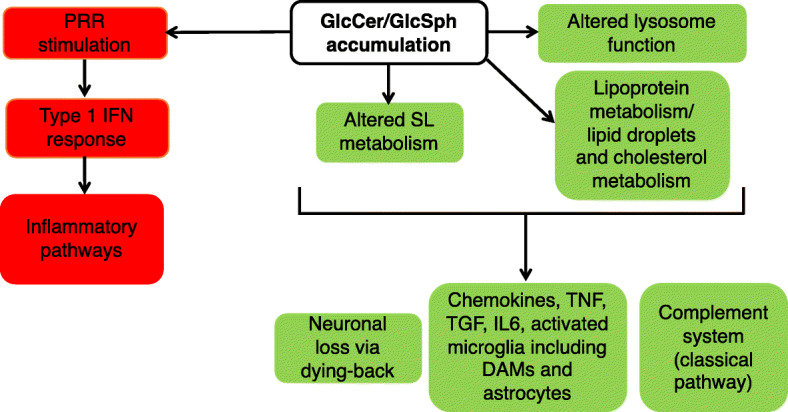
Primary and secondary pathological pathways in nGD. Upon GlcCer/GlcSph accumulation, changes occur in a number of pathways related to altered SL, lipid or lysosomal function, which results in concomitant changes in a number of downstream pathways (*green*), which are classified as primary pathological pathways. IFN-associated pathways (*red*) can be eliminated without effect on mice lifespan, suggesting that they are secondary pathways. For further details, see text

The availability of this data allows us to further interrogate the ~210 genes associated with primary pathology from the current study, a selection of which are listed in Table 3, along with an indication of whether levels of expression of these genes is altered in the SRT study. We propose that genes that were *not* DE in the current study using MyTrMaSt null mice, along with those that *were* DE in the SRT study, are likely to be those that are critically involved in pathophysiology. By way of example, three DEGs in the SL metabolic pathway remained elevated in MyTrMaSt null mice but were reduced by SRT (Table 3); likewise some genes associated with cholesterol metabolism, and the three genes associated with lipoprotein metabolism and lipid droplets, including *Plin4*, whose expression actually increased in MyTrMaSt null mice but reverted to control levels in mice treated with SRT. Lipid droplets have been implicated in neurodegeneration [29, 38], in Parkinson’s disease [39], and in the aging brain [40] implying that they may play a broad role in neurodegenerative diseases. Similarly, lysosomal genes were unaffected in MyTrMaSt null mice but most reverted to control levels after SRT. Since all of these pathways are related, either directly or indirectly to changes in lysosomal SL metabolism, we suggest that they may be coupled to some of the earliest events that occur in nGD pathology, although we cannot determine the precise temporal sequence of events that leads to, or causes changes in their expression (Fig. 5).

As a result of these changes, or concomitant with these changes, a number of inflammatory pathways are activated including those associated with TNF, TGF, IL6, and associated chemokines, along with microglia and astrocyte activation, and upregulation of some components of the complement cascade (Fig. 5). It should be emphasized that the demarcation of genes as associated with primary and secondary pathological pathways is somewhat arbitrary with respect to a number of genes and pathways, with some components of the same pathways appearing in both. This is probably because many genes are components of multiple pathways that impinge upon each other at a number of signaling hubs that are downstream to more than one activator.

Concerning inflammatory pathways that appear associated with primary pathology, two chemokines may play critical roles in nGD, namely *Ccl3* and *Ccl4,* along with other components of TNF signaling, noticeably *Slamf9* and *Cd109*, associated with TGF signaling*. Cd68* and *Mpeg1* may be important for microglia activation, and *Gfap* critical in astrocytosis (Table 3). It is difficult to ascribe precise functions to each of these genes in nGD.

In contrast, functional significance can be advocated for genes associated with a novel microglia type, namely disease-associated microglia (DAM), which have been ascribed roles in Alzheimer’s disease [41] and in several other neurodegenerative diseases [42, 43], including the LSD, mucolipidosis type IV [33]. Several DAM signature genes were upregulated in our study, including *Trem2* and *Tyrobp* (*Dap12*), which remained elevated in MyTrMaSt null mice but were reduced to some extent after SRT. Importantly, Trem2 overexpression attenuates neuroinflammation in Parkinson’s disease [44]. Phospholipids and lipoproteins (including *Apoe* which was reduced to some extent in response to SRT, Table 3) have been identified as ligands for TREM2 in Alzheimer’s disease, which promotes microglia activation and survival. Thus, TREM2 genetic variants, which interfere with this binding, increase the risk of Alzheimer’s disease [45]. Microglia, the only myeloid population in the brain in nGD [46], and its cell surface receptor, TREM2, the principle regulator that transforms microglia from a homeostatic to neuronal disease-associated state, appear critical in nGD pathology. It remains to be elucidated whether the TREM2 or TYROBP signaling is beneficial or detrimental [47].

Another critical gene family appears to be the complement system. The complement system is considered the first line of defense against pathogens, mediates the clearance of immune complexes and regulates inflammatory responses. Our data supports the notion that complement activation in nGD is through activation of the classical pathway [48]. The complement system has previously been implicated in GD, with complement activation suggested to be due to GlcCer accumulation, which leads to the break of tolerance and induction of GlcCer-specific IgG autoantibodies [49, 50].

Finally, three metalloproteases are involved with primary pathology, which may be related to neuronal function. Matrix metalloproteinases (MMPs) and their tissue inhibitors (TIMP) have been implicated in the pathology of Parkinson’s and Alzheimer’s diseases, where MMP can cleave amyloid beta [51]. Similarly, the induction of MT1/2 in the Alzheimer’s disease brain was suggested to be a defense cellular response against inflammatory signals, thus serving a neuroprotective effect [52, 53], though other studies suggest that MT1/2 may have detrimental consequences in amyloid beta clearance [54, 55].

MT1/2 were also implicated in Parkinson’s disease where their elevation was proposed to have a protective role against neurotoxicity [56]. MT1/2 was also unaltered in in MyTrMaSt null mice, suggesting their involvement in GD neuropathology.

## Conclusions

We have differentiated between two types of pathways that are activated in nGD, namely those directly related to disease pathology and those that appear only to be secondarily related (Fig. 5). While this distinction might be somewhat artificial, since individual components of each pathway are activated in the same brains and certainly impact upon each other, our approach should nevertheless help distinguish between pathways that are valid therapeutic targets and those that are not, as well as providing mechanistic insight into how the three major cell types in the brain are affected in this devastating neurological disease.

## Supplementary information

**Additional file 1.** Primers used for RT-PCR

## Data Availability

The RNAseq dataset generated during the current study was deposited in the Gene Expression Omnibus (GEO) database, http://www.ncbi.nlm.nih.gov/geo (accession no. GSE150266).

## Abbreviations

CBE: Conduritol B-epoxide
cGAMP: 2′,3′-cyclic guanosine monophosphate-adenosine monophosphate
cGAS: Cyclic GMP-AMP synthase
DAM: Novel microglia type associated with neurodegenerative diseases
DAMP: Danger-associated molecular patterns
GCase: Acid beta-glucosidase
GD: Gaucher disease
GlcCer: Glucosylceramide
IFN: Interferon
LSD: Lysosomal storage disorder
MAVS: Mitochondrial antiviral signaling protein
MDA5: Melanoma differentiation-associated factor 5
MyD88: Myeloid differentiation factor 88
NOD: Nod-like receptor
PAMP: Pathogen-associated molecular patterns
PRR: Pathogen recognition receptor
RIG: Retinoic acid-inducible gene I
RLR: RIG-I-like receptor
SL: Sphingolipid
STING: Stimulator of IFN genes
TLR: Toll-like receptors
TRIF: TIR-domain-containing adaptor protein-inducing IFN-β
