# Supplementary material for: Mice defective in interferon signaling help distinguish between primary and secondary pathological pathways in a mouse model of neuronal forms of Gaucher disease
Source: J Neuroinflammation. 2020 Sep 7;17:265. doi: 10.1186/s12974-020-01934-x (PMC7487497; doi:10.1186/s12974-020-01934-x)
Supplement: Supplementary file 1 — Additional file 1. Primers used for RT-PCR [file 12974_2020_1934_MOESM1_ESM.docx]

**Table S1.** Primers used for RT-PCR

| Gene name | Primer sequence |
| --- | --- |
| *Hprt* | F: 5′-TGCTCGAGATGTCATGAAGG-3′  R: 5′-AATCCAGCAGGTCAGCAAAG-3′ |
| *Tlr1* | F: 5′-GGACCTACCCTTGCAAACAA-3′  R: 5′-GGTGGCACAAGATCACCTTT-3′ |
| *Tlr2* | F: 5′-TGCTTTCCTGCTGGAGATTT-3′  R: 5′-TGTAACGCAACAGCTTCAGG-3′ |
| *Tlr3* | F: 5′-TTGTCTTCTGCACGAACCTG-3′  R: 5′-CGCAACGCAAGGATTTTATT-3′ |
| *Tlr4* | F: 5′-ACCTGGCTGGTTTACACGTC-3′  R: 5′-CTGCCAGAGACATTGCAGAA-3′ |
| *Tlr5* | F: 5′-GCCACATCATTTCCACTCCT-3′  R: 5′-ACAGCCGAAGTTCCAAGAGA-3′ |
| *Tlr6* | F: 5′-CCAAGAACAAAAGCCCTGAG-3′  R: 5′-TGTTTTGCAACCGATTGTGT-3′ |
| *Tlr7* | F: 5′-GGAAATTGCCCTCGATGTTA-3′  R: 5′-CAAAAATTTGGCCTCCTCAA-3′ |
| *Tlr8* | F: 5′-GAAGCATTTCGAGCATCTCC-3′  R: 5′-GAAGACGATTTCGCCAAGAG-3′ |
| *Tlr9* | F: 5′-ACTGAGCACCCCTGCTTCTA-3′  R: 5′-AGATTAGTCAGCGGCAGGAA-3′ |
| *Tlr11* | F: 5′-TCCTTCCTCTGATTAGCTGTCCTAA-3′  R: 5′-TCCACATAATTTCCACCAACAAGT-3′ |
| *Tlr12* | F: 5′-GCCGCCATTCCAAGCTATC-3′  R: 5′-CTCCACAGTCCGAGGTACAACTT-3′ |
| *Tlr13* | F: 5′-CTTTGTATACCCATGCCTCATCAG-3′  R: 5′-CTTTGTATACCCATGCCTCATCAG-3′ |
| *Rig-i* | F: 5′-CAGACAGATCCGAGACACTA-3′  R: 5′-TGCAAGACCTTTGGCCAGTT-3′ |
| *Mda5* | F: 5′-CGATCCGAATGATTGATGCA-3′  R: 5′-AGTTGGTCATTGCAACTGCT-3′ |
| *Nod2* | F: 5′-CGACATCTCCCACAGAGTTGTAATCC-3′  R: 5′-GGCACCTGAAGTTGACATTTTGC-3′ |
| *cGAS* | F: 5′-GTTCAAACACAAGAAATGCACTG-3′  R: 5′-GCTGACGGAGTACACAATCCT-3′ |
| *Sting* | F: 5′-TGAAAGGCTCTTCATTGTCTCTT-3′  R: 5′-TGGCATCTTCTGCTTCCTAGA-3′ |
| *Irf7* | F: 5′-CAATGGCTGAAGTGAGGGGG-3′  R: 5′-GACCGAAATGCTTCCAGGGT-3′ |
| *Usp18* | F: 5′-CAGGAGTCCCTGATTTGCGT-3′  R: 5′-GGGCTGGACGAAACATCTCA-3′ |
| *F4/80* | F: 5′-TTTCCTCGCCTGCTTCTTC-3′  R: 5′-CCCCGTCTCTGTATTCAACC-3′ |
| *Ccl5* | F: 5′-TGCCTACCTCTCCCTCGCGC-3′  R: 5′-GGCACACACTTGGCGGTTCCT-3′ |
| *Gfap* | F: 5′-TAGTCCAACCCGTTCCTCCA-3′  R: 5′-CCAGTTGTCGACTAGGACCG-3′ |
| *Ccl3* | F: 5′-TTTTGAAACCAGCAGCCTTT-3′  R: 5′-AGTGGGCAGACCCAGCTGGC-3′ |
| *Irf7^1^* | F: 5′-TGGGCAATGACTTGTCAGCAG-3′  R: 5′-CATACCCATGGCTCCAGCTT-3′ |
| *Irg1* | F: 5′-CCTTCTGCTCAGCTTCTCTACT-3′  R: 5′-GGTCTTATGCCACACTGCTT-3′ |
| *C3* | F: 5′-AAGCATCAACACACCCAACA-3′  R: 5′-CTTGAGCTCCATTCGTGACA-3′ |
| *Plin2* | F: 5′-AAGAGCCAGGAGACCATTT-3′  R: 5′-TCCACCCACGAGACATAGA-3′ |
| *Mt1* | F: 5′-CCTCTAAGCGTCACCACGA-3′  R: 5′-GCAGGAGCTGGTGCAAGT-3′ |
| *Gusb* | F: 5′-CCGATTATCCAGAGCGAGTATG-3′  R: 5′-CTCAGCGGTGACTGGTTCG-3′ |
| *CtsD* | F: 5′-CTGAGTGGCTTCATGGGAAT-3′  R: 5′-CCTGACAGTGGAGAAGGAGC-3′ |
| *Cx3cr1* | F: 5′-CAGCATCGACCGGTACCTT-3′  R: 5′-GCTGCACTGTCCGGTTGTT-3′ |
| *C5ar1* | F: 5′-AGACCTGTGCTGTTGTGTCC-3′  R: 5′-GCTTGGATATGTTGGGTTCCATTA-3′ |
| *Serpinf2* | F: 5′-TTCTCCTCAACGCCATCCA-3′  R: 5′-GGTGAGGCTCGGGTCAAAC-3′ |
| *Glycam1* | F: 5′-CCTGCCTGGGTCCAAAGATGAAC-3′  R: 5′-CTGGTGTAGCTGGTGGGAGTGGAC-3′ |
| *Apln* | F: 5′-GGCCTTCTCCGTCTTTGTCG-3′  R: 5′-CCCTCTTGTGCTTCTATCTCTCC-3′ |

^1^ This primer was used for *Irf7* validation.
